# Effect of Disturbance on Population Structure and Regeneration of *Abies fargesii* var. *faxoniana* (Rehder & E.H.Wilson): Mild Disturbance Promotes Growth, Severe Disturbance Leads to Decline

**DOI:** 10.1002/ece3.73942

**Published:** 2026-07-14

**Authors:** Yang Zhao, Bo Li, Wen Yu, Rui Qi, Benqiang Gao, Xuemei Ha, Ting Liu, Yi Li

**Affiliations:** ^1^ College of Forestry Gansu Agricultural University Lanzhou China; ^2^ Forestry Science Institute of Bailongjiang River in Gansu Province Lanzhou China; ^3^ National Positioning Observation and Research Station of Bailongjiang Forest Ecosystem in Gansu Province Zhouqu China; ^4^ College of Earth and Environment Sciences Lanzhou University Lanzhou China

**Keywords:** *Abies faxoniana*, disturbance levels, population structure, regeneration

## Abstract

Plant population structure and regeneration are effected by species traits, environmental conditions, and anthropogenic disturbance. *Abies fargesii* var. *faxoniana* (Rehder & E.H.Wilson), As a major constructive tree species in extreme high‐altitude habitats on the northeastern Qinghai‐Tibetan Plateau, the population structure, regeneration status, and development trends under different disturbance intensities remain unclear. We established plots of *A. faxoniana* natural forests under varying disturbance levels (seven each for mild, moderate, and severe disturbance), investigated population age structure and regeneration characteristics, and analysed population viability and predicted development trends using life tables, survivorship curves, and quantitative methods. The results showed that under light disturbance, the density of individuals and regeneration, and the survival rate were highest; the proportion of young individuals was largest; and the population structure exhibited an inverted ‘J’ shape, a growing population. Under moderate disturbance, the proportion of young individuals decreased, but the population was still growing. Under severe disturbance, the proportion of young individuals decreased significantly, and the population structure became spindle‐shaped, a declining population. An increased level of disturbance led to higher mortality and lower life expectancy. The survival curve of populations under light and moderate disturbance followed Deevey type II (subtype B3), while populations under severe disturbance followed subtype B1. Under light disturbance, the population was stable and exhibited the greatest growth potential, under severe disturbance, growth potential was lowest. Under moderate disturbance, the population structure was most susceptible to change and sensitive to external disturbance. Disturbance significantly effects on the population structure, regeneration, and stability of *A. faxoniana* forests. Under light disturbance, the population structure remains stable with good renewal and significant growth potential. and the population can grow steadily after passing through the bottleneck stage. Under moderate disturbance, growth potential declines. Under severe disturbance, growth potential is severely insufficient, showing a declining trend. Therefore, populations under moderate and especially severe disturbances must be protected more effectively, alongside reasonable disturbance to promote population recovery and stability. Our findings reveal the effects of different disturbance intensities on the population structure and regeneration of *A. faxoniana*, providing a basis for the restoration and disturbance regulation of forest ecosystems in high‐altitude regions.

## Graphical abstract

Disturbance critically affects population structure and stability, and that appropriate disturbance can promote population development. The *Abies faxoniana* population under mild disturbance regenerated well, and the remaining young individuals were able to support population stability and growth, while populations under moderate and severe disturbance require conservation measures and scientifically guided disturbance management to facilitate recovery.

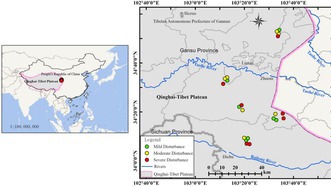

## 1 Introduction

Population structure and population dynamics are fundamental topics in the field of population ecology. Population structure refers to the distribution of individuals of different ages and sizes within a population, reflecting its survival status and developmental trends (Nagel and Cerioni 2023). Population dynamics refer to changing pattern in the size or number of individuals within a population over time and space, reflecting the relationship between the population and its environment (Zhao, Cao, and Li 2020; Zhao, Liu, et al. 2020). Natural regeneration, as a means of self reproduction and recovery in forest ecosystems, directly effects the future structure, function, and biodiversity of forest communities (Li et al. 2020; Wei et al. 2022). Research into population structure, dynamics, and regeneration can objectively reveal the age structure and dynamics of different populations, and the stability and direction of community succession (Zhang and Ru 2010). This research is also holds significant practical importance in guiding the sustainable management of ecosystems (Dang et al. 2010; Zhang and Ru 2010).

The Bailongjiang and Taohe River forest areas are located at the intersection of the Qinghai‐Tibetan Plateau ecological barrier and the Sichuan‐Yunnan Loess Plateau ecological barrier. These regions are a important water conservation and supply zones for the upper and middle reaches of the Yangtze and Yellow Rivers, and home to the largest natural forests of *Picea* and *Abies* in northwest China, and a rich reservoir of biological species. They are recognised as a gene bank for alpine species worldwide and one of the global centers for the origin, distribution, and differentiation of biodiversity. *A. faxoniana*, as the dominant species in this region, is mainly distributed on shady slopes, semi‐shady slopes, and valleys at altitudes of 2800–3800 m, often mixed with *Picea* to form climax communities (Zhao et al. 2024). It plays an irreplaceable role in water conservation, soil retention, and maintaining regional ecological balance. Consequently, the population structure and stability of *A. faxoniana* have received widespread attention. For instance, Tao et al. (2023) found that the age structure of *A. faxoniana* populations is significantly differentiated, with strong regeneration capabilities, but there is a bottleneck in the transition from seedlings to adult trees (Kang et al. 2015). Fencing measures have been demonstrated to significantly improve seedling survival (Zhang et al. 2020), Furthermore, forest gaps can improve mild conditions, thereby promote seedling growth and regeneration (Chen et al. 2016, 2018). However, interspecific competition and environmental stress remain important factors effecting the population structure of *A. faxoniana* (Wang et al. 2019).

In recent years, with the intensification of global climate change and human activities, the stability and continuity of forest structures in high‐altitudes have been continuously disrupted, leading to increasing forest fragmentation (Peng et al. 2023; Kueppers et al. 2017). As a dominant and foundational species in the extreme habitats of high latitude and altitude in the northeastern part of the Qinghai‐Tibet Plateau, the distribution and population structure of *A. faxoniana* have undergone significant changes (Dang et al. 2013, 2014). These changes have severe consequences for population recovery and forest ecosystem functioning, posing a threat to local biodiversity and ecological security. Despite the fact that a considerable number of studies have concentrated on the population structure and natural regeneration of *A. faxoniana* (Kang et al. 2015; Zhang et al. 2020), there remains a paucity of in‐depth research on the specific effects of human disturbance on its population structure. The aim of this study is to analyse the age structure and quantitative changes of *A. faxoniana* populations under different disturbance levels, to answer the following question: (1) How do the population structures of *A. faxoniana* differ under different disturbance conditions? (2) As a foundation species, how do the developmental trends of *A. faxoniana* differ under different disturbance conditions? We hope that the results of this study will provide insights and a theoretical foundation for the sustainable management of forests and the restoration of vegetation in subalpine regions.

## 2 Materials and Methods

### 2.1 Research Zones Natural Overview of the Study Area

This study was conducted in the Zirun Mountain Forest Area in the upper reaches of the Bailongjiang River, and the Dayu Valley, Lu'er Valley, and Yeliguan Forest Areas in the upper reaches of the Taohe River. These 4 study areas are located in Diebu, Zhuoni, and Lintan counties in the Gannan Prefecture (102.18°–104.05°E, 33.39°–35.16°N). The average annual rainfall in these regions is 634–680 mm, annual sunshine duration of 2276–2364 h, and an average annual temperature of 2.3°C–5.8°C. Altitudes range from 1000 to 4300 m, with abundant water resources. The soils are predominantly brown and dark brown, and the forest vegetation consists mainly of dark coniferous forests, with distinct vertical zonation and differences between sunny and shady slopes (Zhao et al. 2024). The dominant tree species in the forest communities include *Abies*, *Picea*, and *Betula*. The area is rich in wildlife and plant resources, and the overall forest quality is high.

### 2.2 Survey and Sampling

From August to September of 2024, we selected representative forest stands of *A. faxoniana* forests in the study area and established 29 standard plots measuring 20 m × 30 m, following the method of Fang et al. (2009). The proportion of *A. faxoniana* in these plots was over 96%. Using the adjacent grid method, with 5 m × 5 m as the basic unit, we recorded the diameter at breast height (DBH), tree height, and crown width of all tree species with DBH ≥ 5 cm within the plots. For saplings and young trees with DBH < 5 cm, we measured and recorded their basal diameter, height, and crown width individually. Each plot was located using GPS, and factors such as latitude, longitude, altitude, and canopy density were recorded.

### 2.3 Classification of Disturbance Levels

There were no records of fire in the study area; all disturbances mentioned in this study are anthropogenic. To quantify these disturbances, we surveyed the number of stumps in each plot, calculated the proportion of stumps, and measured canopy closure (Cao et al. 2022) using a canopy analyser. Additionally, we surveyed the distance from each plot to sites of human activity (including villages and pastures), the distance from the plot to the forest edge, traffic conditions near the plot, and traces of human activity near the plot (including grazing, firewood collection, and domestic waste). Each of these factors was quantified and assigned a value accordingly.

The stump proportion was calculated as follows: stump proportion = number of stumps/(number of stumps + number of individual trees); the higher stump proportion, the greater intensity of historical logging disturbance. Canopy density (CD) was measured using the 5‐point method, and the mean value was taken as the canopy closure of the plot. The current disturbance pressure indicators included the following four variables: distance from the sampling point to human activity sites (far from human activity sites: *L* ≥ 5 km, recorded as 1; moderately far: 2 km ≤ *L* < 5 km, recorded as 2; close: *L* < 2 km, recorded as 3; the closer the distance, the greater the disturbance pressure); traffic conditions around the sampling point (provincial roads or scenic roads, recorded as 3; township roads or mountain trails, recorded as 2; no road, recorded as 1; better traffic accessibility corresponds to greater disturbance pressure); distance from the sampling point to the forest edge (*L* < 0.5 km, recorded as 3; 0.5 km ≤ *L* < 1 km, recorded as 2; *L* ≥ 1 km, recorded as 1; the closer to the forest edge, the greater the disturbance pressure); and disturbance traces around the sampling point (including firewood collection, grazing, and domestic waste traces: 1 type of trace present, recorded as 1; 2 types present, recorded as 2; 3 or more types present, recorded as 3).

**FIGURE 1:**
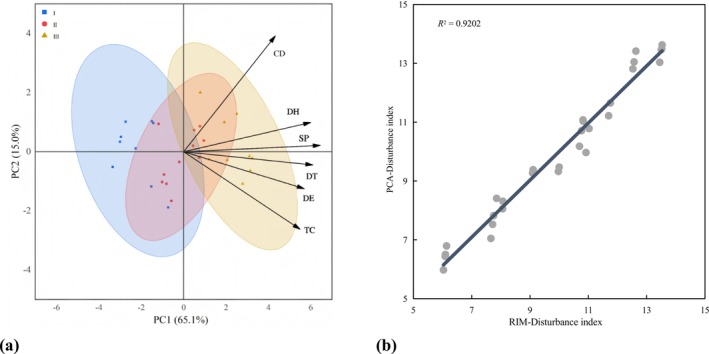
Disturbance index construction.

We used two methods, the Principal Component Analysis (PCA) and Relative Impact Method (RIM) (Sagar et al. 2003), to calculate the comprehensive disturbance index and evaluate the disturbance degree of the sample plots. The disturbance index is calculated using PCA as follows: Stump proportion (SP) represents historical logging disturbance intensity, canopy density (CD) represents forest structural integrity (Li et al. 2002), and human activity intensity represents the current persistent disturbance pressure (including four indicators: distance from the plot to human activity sites (DH), traffic conditions near the plot (TC), disturbance traces around the plot (DT), and distance from the plot to the forest edge (DE)). A total of six factors were subjected to PCA to calculate a comprehensive disturbance index (Figure 1a). The disturbance index is calculated using RIM as follows: For example, plot no. 28 had the smallest stump proportion of 0.024. The stump proportions of adjacent plots 26 and 27 were 0.029 and 0.104, respectively. Therefore, we set the relative disturbance value of plot No. 28 (with the smallest stump proportion) as 1, and the stump proportion values of plots 26 and 27 were 1.167 (0.029/0.024) and 4.242 (0.104/0.024), respectively. This method was used to calculate the stump proportion values for all 29 plots. The relative effect values of the other 5 disturbance factors were also calculated accordingly. Finally, the sum of the relative values of the 6 disturbance factors was taken as the anthropogenic disturbance intensity value.

Finally, referring to the ecological disturbance classification system (Li et al. 2002) and considering the actual conditions of the *A. faxoniana* forest plots, the equal interval method was applied based on the distribution of the comprehensive disturbance index to classify the 29 plots into three disturbance levels: mild disturbance, moderate disturbance, and severe disturbance.

**FIGURE 2:**
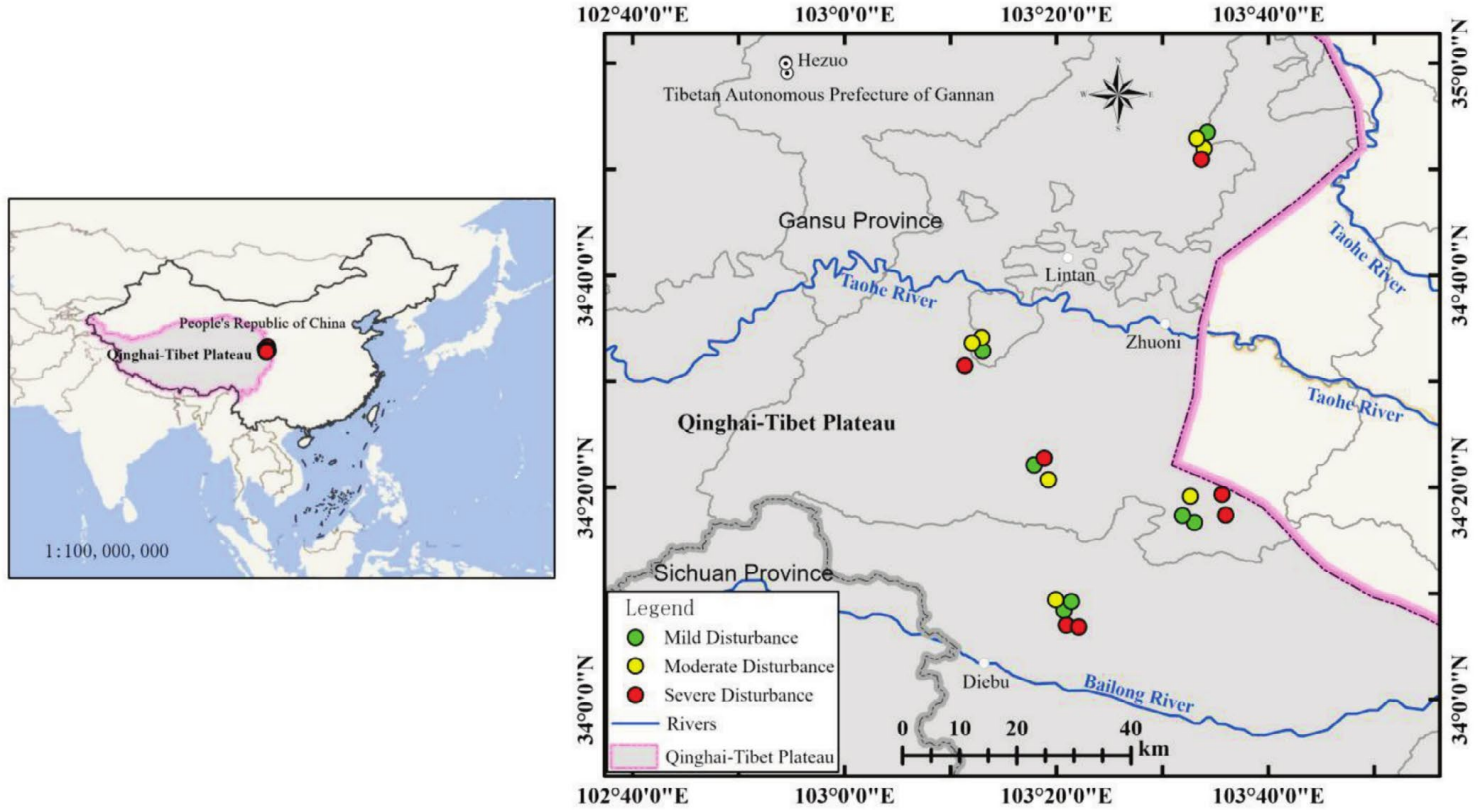
The location of the plots in China.

**FIGURE 3:**
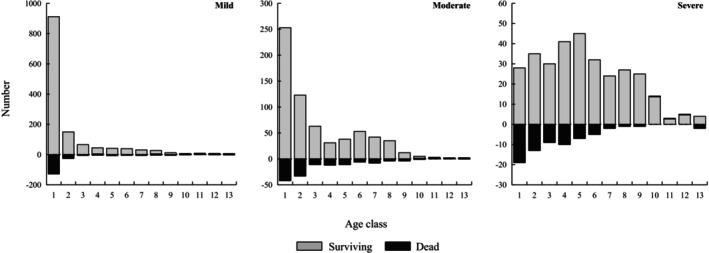
Size class structure of populations.

**FIGURE 4:**
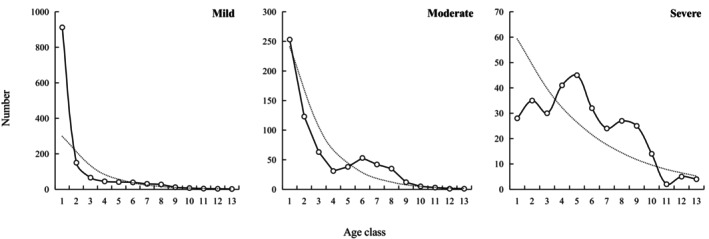
Survival curve of populations.

The PCA evaluation results showed that nine plots were classified as mild disturbance (3, 4, 8, 11, 16, 21, 26, 28, 29), 11 plots as moderate disturbance (1, 2, 5, 6, 7, 9, 10, 17, 18, 19, 20), and nine plots as severe disturbance (12, 13, 14, 15, 22, 23, 24, 25, 27). The RIM evaluation results showed that nine plots were classified as mild disturbance (3, 4, 6, 7, 16, 21, 26, 28, 29), 13 plots as moderate disturbance (1, 2, 5, 8, 9, 10, 11, 17, 18, 19, 20, 22, 27), and seven plots as severe disturbance (12, 13, 14, 15, 23, 24, 25). The two results were found to be highly consistent (*R* ^2^ = 0.9202) (Figure 1b). Consequently, plots with consistent disturbance levels from both methods were selected, i.e., seven plots each for mild disturbance (3, 4, 16, 21, 26, 28, 29), moderate disturbance (1, 2, 10, 11, 18, 19, 27), and severe disturbance (12, 13, 14, 15, 23, 24, 25) (as shown in Figures 2, 3, 4). A total of 21 plots (20 m × 30 m) were involved in this study (Figure 2).

### 2.4 Classification of Size Classes

Based on the principle that the same tree species show consistent responses to the same environmental conditions in terms of age and diameter classes (Forst and Rydin 2000; Wu et al. 2010), we used the space‐for‐time substitution method and size structure analysis to study the population structure of *A. faxoniana*. Age (diameter) classes were divided into intervals of 5 cm based on diameter at breast height (DBH), as follows: Class I (DBH < 5 cm), Class II (5 cm ≤ DBH < 10 cm), Class III (10 cm ≤ DBH < 15 cm), and so on. According to the characteristics of the survey data, all surveyed *A. faxoniana* were classified into 13 size classes. A population structure diagram of *A. faxoniana* was then plotted, with size classes on the vertical axis and the corresponding number of individuals on the horizontal axis.

### 2.5 Life Table and Survival Curve

We constructed a static life table based on the actual number of survivors in each size class, with size classes corresponding to size classes. The construction and calculation methods are referenced from Liu et al. (2023) and Harcombe (1987). Using size class as the *x*‐axis and the actual number of surviving individuals (*A*_*x*_) as the *y*‐axis, we plotted the survival curve for the *A. faxoniana* population. The relationship between number of survivors and size class was fitted using both exponential and power functions. The goodness of fit of these models was determined using the coefficient of determination and *F*‐test values (Silvertown 1982). If the exponential equation *N*_*x*_ = *N*_0_ *e* ^−*bx*^ provided a better fit, the survival curve was classified as Deevey‐II type; if the power function *N*_*x*_ = *N*_0_ *x* ^*−b*^ provided a better fit, the survival curve was classified as Deevey‐III type.

### 2.6 Population Dynamics Analysis

The quantitative analysis of population dynamics is based on the dynamic index of changes in the number of individuals between two adjacent size classes within a population (*V*_*n*_), the dynamic index of changes in the quantitative structure of the entire population age distribution (*V*_*pi*_), and the dynamic index of the population age structure considering future external disturbances ($V_{\mathrm{pi}}^{\prime }$). These indices allow an objective and precise quantitative comparison and evaluation of the structural dynamics of a population (Chen 1998). The calculation formulae are as follows:

(1) $$V_{n}=\frac{S_{n}-S_{n+1}}{\max\;\left(S_{n},S_{n+1}\right)}\times 100\%$$

(2) $$V_{\mathrm{Pi}}=\frac{1}{\sum_{n=1}^{k-1}S_{n}}\sum_{n=1}^{k-1}\left(S_{n}V_{n}\right)$$

where *V*_*n*_ is the dynamic change in the number of individuals from size class *n* to (*n* + 1), and *V*_*pi*_ is the dynamic index of the overall population structure change. *S*_*n*_ and *S*_*n*_ + 1 are the number of individuals in the *n*th and (*n* + 1)th size class, respectively. *k* is the number of size classes in the population. The functions max/min (…) take the maximum and minimum values from the sequence within the parentheses. *V*_*n*_ Є [−1, 1], where positive, negative, and 0 values of *V*_*n*_ indicate growth, decline, and stability in the population structure between two adjacent size classes (or the overall size structure of the population), respectively. *V*_*pi*_ is only applicable for comparing population structure dynamics without considering future external environmental disturbances. When future external disturbances are considered, the dynamics of the population age structure ($V_{\mathrm{pi}}^{\prime }$) also depends on the number of size classes *k* and the number of individuals in each size class *S*_*n*_. Therefore, Equation (2) is revised as follows:

(3) $$V_{\mathrm{pi}}^{\prime }=\frac{\sum_{n=1}^{k-1}\left(S_{n}V_{n}\right)}{\min\left(S_{1},S_{2},S_{3},\ldots ,S_{k}\right)k\sum_{n=1}^{k-1}S_{n}}$$

(4) $$p_{\max}=\frac{1}{k\;\min\left(S_{1},S_{2},S_{3},\ldots ,S_{k}\right)}$$

where $V_{\mathrm{pi}}^{\prime }$ can also serve as an indicator to measure the sensitivity of population structure dynamics to stochastic disturbances. The dynamic relationships reflected by positive, negative, or 0 values of *V*_*pi*_ and $V_{\mathrm{pi}}^{\prime }$ are consistent with those of *V*_*n*_. *p* represents the risk probability that the population bears due to external random disturbances. Only when the value of *p* is at its maximum does it exert the greatest effect on the population dynamics *V*_*pi*_.

### 2.7 Time Series Model Prediction

We used the method of once average moving in time series analysis to predict the dynamics of the *A. faxoniana* population for the next 2, 4, 6, and 8 size classes. The model is presented as follows:

(5) $$M_{t}^{1}=\frac{1}{n}\sum_{k=t-n+1}^{t}X_{k}$$

where *n* is the number of future years to be predicted; *t* denotes the size class; $M_{t}^{1}$ is the population survival number at size class *t* in the future *n* years, which is the average of the recent *n* observations at time *t*, known as the moving average of the *n*th cycle; and *X*_*k*_ is the current population survival number at size class *k*.

## 3 Results and Analysis

### 3.1 Population Regeneration and Survival Status

After checking normality with the Shapiro–Wilk test and homogeneity of variances with Levene's test, all variables did not violate satisfy the assumptions for parametric methods (*p* > 0.05). Differences among disturbance levels were examined using one‐way ANOVA, followed by Tukey HSD post hoc comparisons, and the significance threshold was set at *α* = 0.05. As shown in Table 1, stand density and regeneration status of the *A. faxoniana* forest varied considerably under different disturbance levels. Stand density, seedling number, regeneration density, frequency, and survival rate all followed the order: mild disturbance > moderate disturbance > severe disturbance. Specifically, total stand density, seedling number, regeneration density, and frequency were significantly higher under mild disturbance than under moderate disturbance (*p* < 0.001), and significantly higher under moderate disturbance than under severe disturbance (*p* < 0.001). However, large tree density and seedling survival rate showed no significant differences between mild and moderate disturbance, but both were significantly higher under mild and moderate disturbance than under severe disturbance (*p* < 0.001). Therefore, disturbance significantly affects stand density and all regeneration (*p* < 0.001). Under mild disturbance, population regeneration is good, as disturbance increases, stand density and regeneration capacity decline.

**TABLE 1:** Forest regeneration and survival status.

Disturbance level	Total density/plant hm^−2^	Density of large trees/plant hm^−2^	Number of seedlings/plant	Regeneration density/plant hm^−2^	Seedling survival rate/%	Frequency/%
Mild	4052.38 ± 270.67a***	1814.29 ± 215.36a***	131.57 ± 24.53a***	2280.95 ± 306.93a***	74.34 ± 7.40a**	68.79 ± 3.85a***
Moderate	2266.67 ± 125.51b***	1597.62 ± 130.29a***	44.57 ± 6.75b***	773.81 ± 122.47b***	72.07 ± 6.69a**	51.33 ± 3.75b***
Severe	1133.33 ± 82.13c***	1006.52 ± 94.62b***	5.71 ± 0.99c***	133.33 ± 17.82c***	38.14 ± 9.60b**	23.33 ± 2.82c***

*Note:* Different lowercase letters indicate significant differences between respective means. *p < 0.05, **p < 0.01, ***p < 0.001.

### 3.2 Size Class Structure of Populations

As shown in Figure 3, we investigated populations of *A. faxoniana* under mild, moderate, and severe disturbance, and recorded 1532, 793, and 382 individuals, respectively. The numbers of surviving individuals were 1339, 661, and 313, with overall survival rates of 87.40%, 83.35%, and 81.94%, respectively. Under mild and moderate disturbance, young individuals accounted for the largest proportion of surviving individuals, reaching 68.26% and 38.39%, respectively. Under severe disturbance, young individuals accounted for only 8.97% of the surviving population, while the 5th size class had the highest proportion at 14.42%. The proportions of dead individuals relative to the total surveyed population were 12.60%, 16.65%, and 18.06%, respectively, and the mortality rates of young individuals were 12.30%, 14.24%, and 40.43%, respectively. Thus, under severe disturbance, the overall mortality rate of the population and the mortality rate of juvenile individuals are highest. Among dead individuals, young individuals accounted for the largest proportion, representing 66.32%, 31.82%, and 27.54% of dead individuals, respectively. This indicates that under mild disturbance, mortality mainly affects young individuals, whereas severe disturbance not only impacts young individuals but also increases mortality among adult individuals.

Overall, under mild disturbance, the number of the population surviving individuals declines with increasing age. Individuals in the juvenile and young size classes (1–2 years) account for nearly 80% of total deaths; after the second size class, mortality drops sharply. The population structure exhibits a typical inverted ‘J’ shape, indicating a growing population. Compared to mild disturbance, under moderate disturbance, the proportion of juvenile and young individuals in the population declines; dead individuals account for over 56% of the total mortality, while deaths among individuals aged 1–5 years exceed 5%. The overall population structure approaches an inverted ‘J’ shape and is also a growth type population. Under severe disturbance, the population structure occurred significant changes; the number and proportion of surviving individuals in the juvenile and young size classes declined markedly, while the mortality rate among individuals in all the first 5 size classes exceeded 10%. The overall structure of the surviving population approached a spindle shape and is a declining population.

### 3.3 Population Survival Process

#### 3.3.1 Static Life Table

As shown in Table 2, under different disturbance levels, the number of individuals in each size class of the *A. faxoniana* population varies greatly. Under mild and moderate disturbance, the population as a whole exhibits a decreasing trend in individual numbers with increasing size class. Under moderate disturbance, there are some fluctuations in size classes 5–8, but the overall trend remains unchanged. Under severe disturbance, the population structure changes dramatically, with fewer individuals in the younger size classes and a larger proportion in the middle size classes.

**TABLE 2:** Static life table of populations.

Disturbance level	Age class	*A*_*x*_	*l*_*x*_	*lnl*_*x*_	*d*_*x*_	*q*_*x*_	*L*_*x*_	*T*_*x*_	*e*_*x*_	*K*_*x*_	*S*_*x*_
Mild	1	912	1000	6.908	835.526	0.836	582.237	967.105	0.967	1.805	0.164
	2	150	164	5.103	92.105	0.560	118.421	384.868	2.340	0.821	0.440
	3	66	72	4.282	23.026	0.318	60.855	266.447	3.682	0.383	0.682
	4	45	49	3.899	4.386	0.089	47.149	205.592	4.167	0.093	0.911
	5	41	45	3.806	2.193	0.049	43.860	158.443	3.524	0.050	0.951
	6	39	43	3.756	8.772	0.205	38.377	114.583	2.679	0.230	0.795
	7	31	34	3.526	4.386	0.129	31.798	76.206	2.242	0.138	0.871
	8	27	30	3.388	16.447	0.556	21.382	44.408	1.500	0.811	0.444
	9	12	13	2.577	5.482	0.417	10.417	23.026	1.750	0.539	0.583
	10	7	8	2.038	3.289	0.429	6.031	12.610	1.643	0.560	0.571
	11	4	4	1.478	1.096	0.250	3.838	6.579	1.500	0.288	0.750
	12	3	3	1.191	1.096	0.333	2.741	2.741	0.833	0.405	0.667
	13	2	2	0.785							
Moderate	1	253	1000	6.908	513.834	0.514	743.083	2110.672	2.111	0.721	0.486
	2	123	486	6.187	237.154	0.488	367.589	1367.589	2.813	0.669	0.512
	3	63	249	5.518	126.482	0.508	185.771	1000.000	4.016	0.709	0.492
	4	31	123	4.808	−27.668	−0.226	136.364	814.229	6.645	−0.204	1.226
	5	38	150	5.012	−59.289	−0.395	179.842	677.866	4.513	−0.333	1.395
	6	53	209	5.345	43.478	0.208	187.747	498.024	2.377	0.233	0.792
	7	42	166	5.112	27.668	0.167	152.174	310.277	1.869	0.182	0.833
	8	35	138	4.930	90.909	0.657	92.885	158.103	1.143	1.070	0.343
	9	12	47	3.859	27.668	0.583	33.597	65.217	1.375	0.875	0.417
	10	5	20	2.984	7.905	0.400	15.810	31.621	1.600	0.511	0.600
	11	3	12	2.473	3.953	0.333	9.881	15.810	1.333	0.405	0.667
	12	2	8	2.068	3.953	0.500	5.929	5.929	0.750	0.693	0.500
	13	1	4	1.374							
Severe	1	28	1000	6.908	−250.000	−0.250	1125.000	10571.429	10.571	−0.223	1.250
	2	35	1250	7.131	178.571	0.143	1160.714	9446.429	7.557	0.154	0.857
	3	30	1071	6.977	−392.857	−0.367	1267.857	8285.714	7.733	−0.312	1.367
	4	41	1464	7.289	−142.857	−0.098	1535.714	7017.857	4.793	−0.093	1.098
	5	45	1607	7.382	464.286	0.289	1375.000	5482.143	3.411	0.341	0.711
	6	32	1143	7.041	285.714	0.250	1000.000	4107.143	3.594	0.288	0.750
	7	24	857	6.754	−107.143	−0.125	910.714	3107.142	3.625	−0.118	1.125
	8	27	964	6.871	71.429	0.074	928.571	2196.429	2.278	0.077	0.926
	9	25	893	6.794	392.857	0.440	696.429	1267.857	1.420	0.580	0.560
	10	14	500	6.215	428.571	0.857	285.714	571.429	1.143	1.946	0.143
	11	2	71	4.269	−107.143	−1.500	125.000	285.714	4.000	−0.916	2.500
	12	5	179	5.185	35.714	0.200	160.714	160.714	0.900	0.223	0.800
	13	4	143	4.962							

*Note:* *A*_*x*_, Number of original survivors; *l*_*x*_, standardised survivals; *d*_*x*_, standardised deaths; *q*_*x*_, mortality rate; *L*_*x*_, survived individuals of the interval from *x* to *x* + 1; *T*_*x*_, total number of individuals from level *x* to greater than level *x*; *e*_*x*_, life expectancy; *K*_*x*_, killing rate; *S*_*x*_, survival rate.

Under mild disturbance, the population experiences its first mortality peak in 1–2 size classes, where interspecific competition is intense, and life expectancy is lowest. Over the next 4 size classes, mortality gradually decreases, and life expectancy gradually increases, peaking in the 4th size class. At this stage, the population environment is stable, which is most conducive to population stability and development. In the 5–6 size classes, mortality begins to rise with fluctuations, and life expectancy shows a gradual downward trend; in the 8th and 10th size classes there are two mortality peaks, and life expectancy gradually stabilizes, indicating that the population has entered a stable development stage.

Under moderate disturbance, the population maintains a very high mortality in 1–3 size classes and experiences its first mortality peak. Life expectancy gradually increases, peaking in the 4th size class. 4–5 size classes, possibly due to disturbance, the number of individuals in the younger size classes decreases, leading to a negative mortality rate. At this point, life expectancy reaches its highest value. Subsequently, mortality gradually increases with fluctuations, reaching a second peak in the 8th size class. After this, mortality shows a gradual downward trend, and life expectancy decreases with fluctuations. After the 8th size class, the population gradually stabilises, indicating that adult trees are less affected by disturbances. The trends in life expectancy under mild and moderate disturbance are similar for the *A. faxoniana* population.

Under severe disturbance, population mortality fluctuates greatly with increasing size class. Negative mortality values occur in size classes 1–2, 3–4, 7–8, and 11–12, due to a reduction in the number of individuals in smaller size classes. Life expectancy is highest at the seedling stage. As the size class increases, life expectancy shows a gradually decreases trend amid fluctuations, indicating that the population and its living environment are unstable and not conducive to development.

#### 3.3.2 Survival Curve

According to Figure 4 and Table 3, under mild, moderate, and severe disturbance, the *R* ^2^ and *F* values of the exponential function model for the survival curve of the *A. faxoniana* population exceed those of the power function model. Therefore, the survival curves of the population all conform to the Deevey‐II type, indicating a high mortality among young individuals. However, for the *A. faxoniana* population under severe disturbance, the number of young individuals is fewer than that of middle‐aged individuals. Thus, although its survival curve is the same type as those under mild and moderate disturbance, there are still differences.

**TABLE 3:** The equation of survival curve for populations.

Disturbance level	Survival curve	Survival curve type
Mild	*Y* = 487.819*e* ^−0.961*x*^, *R* ^2^ = 0.917, *F* = 132.787, *p* < 0.0001	Deevey‐II
	*Y* = 1032.580*x* ^−0.949^, *R* ^2^ = 0.891, *F* = 99.499, *p* < 0.0001	
Moderate	*Y* = 368.797*e* ^*−*0.945*x*^, *R* ^2^ = 0.884, *F* = 92.459, *p* < 0.0001	Deevey‐II
	*Y* = 572.133*x* ^−0.860^, *R* ^2^ = 0.715, *F* = 31.174, *p* < 0.0001	
Severe	*Y* = 72.566*e* ^−0.791*x*^, *R* ^2^ = 0.592, *F* = 18.415, *p* < 0.001	Deevey‐II
	*Y* = 70.320*x* ^−0.614^, *R* ^2^ = 0.320, *F* = 6.641, *p* < 0.026	

The type II curve is categorised into three subtypes: the curves of subtypes B_1_ and B_3_ fluctuate around the diagonal line, with subtype B_1_ showing significant differences in survival rates in different periods, subtype B_2_ is the standard diagonal type with similar mortality rates in all ages, and subtype B_3_ has a higher mortality in the young stage and a lower mortality in adulthood (Lu et al. 2013; Zhao, Cao, and Li 2020; Zhao, Liu, et al. 2020). Combined with Figure 4, it can be seen that under mild and moderate disturbance, the survival curve of the *A. faxoniana* population approaches the B_3_ subtype of the Deevey‐II type curve, under severe disturbance, the survival curve approaches the B_1_ subtype of the Deevey‐II type curve, which is consistent with the results of the population structure and life table analysis.

### 3.4 Population Dynamics Analysis and Time Series Model Prediction

#### 3.4.1 Quantitative Analysis of Population Dynamics

As shown in Table 4, under different disturbance levels, the dynamic index of adjacent size classes of the *A. faxoniana* population varies differently. Under mild disturbance, the dynamic index of adjacent size classes (*V*_*n*_) is greater than 0 for all classes, and the dynamic index of overall population quantity change (*V*_*pi*_) is also greater than 0, indicating that the population structure is stable and in a steady state of growth. Under moderate disturbance, the dynamic index of adjacent size classes fluctuates (*V*_*n*_ < 0) between the 4–5 and 5–6 size classes, showing local declines, but the overall population dynamics remain stable and in a growth state (*V*_*pi*_ > 0). Under severe disturbance, the dynamic index of adjacent size classes fluctuates multiple times (*V*_*n*_ < 0), indicating population instability. The dynamic index of quantitative change of the overall population (*V*_*pi*_) is close to 0, indicating that although the population is generally in a growth state, its growth potential is limited by an insufficient number of young individuals.

**TABLE 4:** Dynamic index of dominant populations in tree layer.

Age class	Dynamic index class	Dynamic index		
		Mild disturbance	Moderate disturbance	Severe disturbance
1–2	*V*_1_	0.836	0.514	−0.200
2–3	*V*_2_	0.560	0.488	0.143
3–4	*V*_3_	0.318	0.508	−0.268
4–5	*V*_4_	0.089	−0.184	−0.089
5–6	*V*_5_	0.049	−0.283	0.289
6–7	*V*_6_	0.205	0.208	0.250
7–8	*V*_7_	0.129	0.167	−0.111
8–9	*V*_8_	0.556	0.686	0.074
9–10	*V*_9_	0.500	0.545	0.440
10–11	*V*_10_	0.500	0.400	0.857
11–12	*V*_11_	0.333	0.667	−0.600
12–13	*V*_12_	0.000	0.000	0.200
*V*_*pi*_		0.681	0.908	0.099
$V_{\mathrm{pi}}^{\prime }$		0.052	0.070	0.003
*p*_max_		0.038	0.077	0.036

*Note:* *V*_*n*_, quantitative dynamics index of adjacent size class; *V*_*pi*_, quantitative dynamics index of population; $V_{\mathrm{pi}}^{\prime }$, quantitative dynamics index of population by external disturbance; *p*_max_, the maximum of probability in random disturbance.

When considering external random disturbances, under different disturbance levels, the dynamic index of the population age structure ($V_{\mathrm{pi}}^{\prime }$) is greater than 0, indicating that the population still exhibits growth structural dynamics. However, under severe disturbance, $V_{\mathrm{pi}}^{\prime }$ (0.003) approaches 0, indicating that the population has minimal growth potential. The maximum probability of the population being affected by external random disturbance risks is *p* (moderate) *> p* (mild) > *p* (severe), indicating that the probability of the population being affected by external random disturbances is small, all below 10%. However, under severe disturbance, the population structure is severely damaged, and it would not change significantly if disturbed again. In contrast, under moderate and mild disturbance, the population type is more likely to change when disturbed again, with the moderate disturbance scenario having the highest probability of being affected by external disturbance.

#### 3.4.2 Time Series Model Prediction

Based on the actual number of survivors, we used the one‐step moving average method to predict the number of surviving individuals in each size class of the *A. faxoniana* population under different disturbance levels after 2, 4, 6, and 8 size classes. As shown in Table 5, under mild disturbance, the number of individuals in each size class of the *A. faxoniana* population shows an increasing after 2, 4, 6, and 8 size classes. Under moderate disturbance, the population only declines at size classes 6–7, while all other size classes exhibit an increasing trend. Under severe disturbance, the population shows a decline at size classes 4–8. Overall, after 8 size classes, the population sizes under mild, moderate, and severe disturbance increase by factors of 5.65, 4.04, and 2.05, respectively. This indicates that population growth potential is greatest under mild disturbance and smallest under severe disturbance, which is consistent with the results of the quantitative analysis of population dynamics.

**TABLE 5:** Time sequence prediction of number dynamics population.

Mild disturbance					Moderate disturbance					Severe disturbance				
*M*_0_	*M*_2_	*M*_4_	*M*_6_	*M*_8_	*M*_0_	*M*_2_	*M*_4_	*M*_6_	*M*_8_	*M*_0_	*M*_2_	*M*_4_	*M*_6_	*M*_8_
912					253					28				
150	531				123	188				35	32			
66	108				63	93				30	33			
45	56	293			31	47	118			41	36	34		
41	43	76			38	35	64			45	43	38		
39	40	48	209		53	46	46	94		32	39	37	35	
31	35	39	62		42	48	41	58		24	28	36	35	
27	29	35	42	164	35	39	42	44	80	27	26	32	33	33
12	20	27	33	51	12	24	35	36	50	25	26	27	32	32
7	10	19	26	34	5	9	24	31	35	14	20	23	28	30
4	6	13	20	26	3	4	14	25	27	3	9	17	21	26
3	4	7	14	21	2	3	6	17	24	5	4	12	16	22
2	3	4	9	16	1	2	3	10	19	4	5	7	13	17

*Note:* *M*_0_, number of original survival; *M*_2_, *M*_4_, *M*_6_, *M*_8_, number of every size class after 2, 4, 6, 8 size class time.

From the above analysis, we conclude that the growth of each size class ultimately depends on the number of seedlings. Therefore, the higher the proportion of seedlings, the greater the growth potential. Considering only the number and survival status of seedlings, under mild and moderate disturbance, the population has the highest seedling proportion and health rate. As young trees grow, the number of middle‐aged and adult individuals will gradually increase and the population structure becomes more reasonable. However, under severe disturbance, the population structure is damaged, with an insufficient number of seedlings and a high mortality rate. This leads to insufficient momentum for population growth or even decline. Although this method's predictions show population growth, the population will stop growing or show a declining trend if seedlings are not replenished in time. Therefore, we believe that time series prediction has limitations for populations under severe disturbance.

## 4 Discussion

### 4.1 Population Regeneration and Structural Characteristics

This study shows that the individual density of *A. faxoniana* trees and various regeneration indicators decreases significantly with increasing disturbance level. Under mild disturbance, the density of individual trees, the density of regeneration and the number of regenerations, as well as the survival rate, are all at their highest. As disturbance level increases, the overall mortality rate of the population rises. Compared with mild disturbance, the proportion of young individuals decreases under both moderate and severe disturbance. Under moderate disturbance, although the population structure changes to some extent, the overall trend remains unchanged. However, severe disturbance disrupts the population structure, resulting in a severe lack of young individuals and leading to a declining trend. Study by Wang et al. (2024) indicates that young individuals, i.e., forest regeneration are fundamental to population survival and development, and changes in them inevitably effect population structure. The survival of young individuals determines the success or failure of population regeneration (Wang et al. 2015). However, the stability of middle‐aged and adult trees also plays an important role in maintaining the stability of the entire population and community (Liu et al. 2023; Zhang and Ru 2010). Not only does disturbance effect the growth of young individuals, it also has an impact on middle‐aged and adult trees that cannot be ignored (Wang et al. 2024). In this study, as disturbance levels, the mortality rate of middle‐aged and adult trees increases. Under severe disturbance, they account for 53.62% of total mortality. Generally, young individuals are the most sensitive to disturbance (Hammond et al. 2022). Therefore, mild disturbance mainly effects young individuals; under moderate disturbance, the population living environment changes, effecting both young and adult individuals. Severe disturbance, such as logging, directly reduces the number of middle‐aged and adult trees, damaging population structure and habitat, and also destroys the microenvironments for seedling establishment and growth (Martin 2024). Furthermore, reducing the number of parent trees due to logging decreases natural seed dispersal, effects regeneration sources, alters population development trends, or leads to changes in community type (Sun et al. 2008). Therefore, when disturbance is excessive, its effects are persistent (Dang et al. 2014); even short term disturbance can have long lasting effects and require a long time to recover (Zhang et al. 2014). When such disturbance persists, the reduction of young individuals will inevitably effect adult trees, leading to a gap in the population age structure and even population extinction.

### 4.2 Population Dynamics and Development Trends

The results of life table analysis showed that, under mild disturbance, population mortality decreased rapidly in size classes 1–5, and then increased with fluctuations after size class 5. Under moderate disturbance, mortality rates were relatively high in size classes 1–3, negative values appeared in size classes 4–5, and then mortality increased with fluctuations after size class 5. Life expectancy increased rapidly in size classes 1–4, peaked at size class 4, and then showed a decreasing trend after size class 4. Under severe disturbance, population mortality fluctuated greatly, with multiple negative values, and life expectancy was highest in the early size classes, followed by a gradual decreasing trend. It is generally believed that the survival status of a population is effected by both internal competition and external disturbance (Nagel and Cerioni 2023; Zhao et al. 2024; Kang and Li 2022). In this study, under mild disturbance, the *A. faxoniana* population exhibited high individual density and high regeneration density, as well as a high regeneration survival rate. Therefore, we speculate that its survival status is mainly effected by intraspecific competition. Under moderate disturbance, the density of individuals in the population, as well as the number and density of seedlings, decreased significantly. However, the density of large trees and the survival rate of seedlings were not significantly effected. Thus, the population is effected by both intraspecific competition and external disturbance. Moreover, under moderate disturbance, although the number of seedlings decreased, they still maintained a considerable advantage. The population survival curve was the same type as that under mild disturbance, both conforming to the Deevey II Type curve, further demonstrating that the population structure type had not yet changed under moderate disturbance. However, under severe disturbance, a substantial reduction in young individuals altered the population's survival status, manifested as fewer individuals in smaller diameter classes than in larger diameter classes, leading to negative mortality values with large fluctuations. This further indicates that disturbance has caused the population structure to become unbalanced, i.e., a declining trend. The density, regeneration density, and survival rate of individual trees were all significantly lower than those under mild and moderate disturbances. Therefore, the population is primarily effected by external disturbance, and the severe damage to population structure and habitat leads to a decrease in life expectancy (Zhao, Cao, and Li 2020; Zhao, Liu, et al. 2020). Under such conditions, we believe that the population's survival curve can no longer accurately reflect its actual survival status and trend.

Some studies suggest that for populations with abundant young individuals (e.g., most coniferous forests), the weak competitive ability of seedlings and limitations of space and resources constitute a ‘bottleneck’ that prevents young individuals from successfully transitioning into the next size class, and population regeneration and development (Fu et al. 2010). The number of effective seedlings that can successfully grow into higher size classes (Gao et al. 2017; Wei et al. 2022) directly effects the regeneration rate and structural stability of the population (Tang et al. 2024). Life expectancy indicates the probability of survival of tree populations or individuals at the current size class or the next stage; the higher the life expectancy, the greater the likelihood of tree survival (Zhang et al. 2024; Zhao, Cao, and Li 2020; Zhao, Liu, et al. 2020). In this study, the *A. faxoniana* population under mild disturbance exhibited good regeneration and high regeneration density. Under moderate disturbance, however, mortality remained high in size classes 1–4, and during these size classes, life expectancy increased, reaching its maximum at size class 4. Therefore, we infer that under mild disturbance, size classes 1–2 represent the population bottleneck period, whereas moderate disturbance significantly reduces the number and density of individual trees and regeneration, prolonging the population bottleneck period (size classes 1–4). Under severe disturbance, the population structure and habitat are severely damaged, the number of young individuals is insufficient, and the population is maintained mainly by a small number of middle‐aged and older individuals. Moreover, the effects of severe disturbance are long‐term, leading to a continuous decline in life expectancy, indicating that the population structure has become highly unstable. Consequently, any size class could become a sensitive period or bottleneck period for the population.

Quantitative dynamics analysis showed that under mild disturbance, all size classes of the *A. faxoniana* population exhibited a growth type (*V*_*n*_ > 0), and the number of individual trees increased nearly 6 fold after 8 size classes. Under moderate disturbance, although the population experienced a temporary decline, the overall growth trend remained unchanged, with the number of individuals increasing more than 4 fold after 8 size classes. Under severe disturbance, the quantitative dynamics index (*V*_*n*_) fluctuated greatly, and the population's growth potential was extremely low. Based on previous studies (Zhao et al. 2018, 2024; Zhao, Cao, and Li 2020; Zhao, Liu, et al. 2020), we believe that both population quantitative dynamics analysis and time series modelling predict population dynamics and future development trends based on the current population structure, especially the current number of young individuals. Therefore, their results are significantly affected by external disturbance (Wang et al. 2015). If the young individuals of a population are destroyed and the structural integrity is lost, the growth trend will decline in subsequent size classes due to the lack of a seedling and sapling base (Zhao, Cao, and Li 2020; Zhao, Liu, et al. 2020), rendering the prediction of population development trends practically meaningless. Thus, under mild disturbance, the analysis results are reliable; once the disturbance level is high enough to damage the population structure, the results lose their reference value. However, under real‐world conditions, disturbance is unavoidable. Therefore, to ensure population growth, it is necessary to strengthen population protection and mitigate disturbance.

### 4.3 Reflections on the Current Status and Management of Community Survival

It is worth mentioning that *A. faxoniana*, as a dominant and major constructive species in the high‐altitude areas of southern Gansu Province. It is widely distributed in the upper reaches of the Bailongjiang and Taohe River. Together with *Picea purpurea* and other species, it forms climax communities that maintain regional ecological balance and dominate the direction of forest succession in this area. However, due to years of logging and disturbance, its forest resources total have declined, and the community structure has become unbalanced. Through this investigation, we found that although the implementation of the Natural Forest Protection Program and the deepening of ecological civilization construction in recent years have gradually improved the community environment and ecosystem structure, the overall *A. faxoniana* forest community shows a trend of recovery. The imbalance in population structure caused by past disturbances, coupled with climatic constraints at mid‐ to high‐altitudes that result in slow tree growth (Zhao et al. 2024), as well as ongoing local disturbances, have led to extremely slow population and community recovery. The restoration process remains lengthy, and the overall trend of vegetation degradation in high‐altitude areas has not yet been fundamentally reversed.

However, the continuous development of economic society makes disturbance unavoidable. It is undeniable that disturbance has become a major factor affecting plant population structure and survival status (Kang et al. 2019). Nevertheless, disturbance also has positive ecological significance, which is mainly reflected in its level and direction (Zhu and Liu 2004; Sun et al. 2008; Chen and Fu 2000). Reasonable disturbance can promote population development toward the target direction (Chen and Fu 2000), such as in sustainable forest management (Liu et al. 2015), reducing the number of individual trees or improving forest structure can reduce competition and benefit the growth of surviving individuals. However, persistent or severe disturbance can seriously affect the health and stability of populations, leading to irreversible changes in successional trends or community types. If such disturbances are not eliminated or mitigated, they would be extremely detrimental to ecological stability. For populations with a large number of young individuals (such as *Picea* and *Abies*), the long‐standing issue of high juvenile mortality in high‐altitude areas remains unresolved. Nevertheless, this study observes that under mild disturbance, the *A. faxoniana* population regenerates well. Even though juvenile mortality is high, the number of surviving effective individuals is sufficient to support the population's sustainable development. Therefore, we believe that populations that have experienced moderate or severe disturbance can also gradually restore their structure through proper management and by relying on their own regeneration capacity, provided that the level or mode of disturbance is appropriate. However, this recovery process is lengthy and arduous.

## 5 Conclusion

Under different disturbance levels, the stand density and regeneration of the *A. faxoniana* forest differ significantly, and the population age structure exhibits different types. Under light disturbance, the population regenerates well, with a high proportion of young individuals, and the age structure is typically an inverted J‐shape. Under moderate disturbance, the proportion of young individuals decreases, but the overall structure remains inverted J‐shaped. Severe disturbance alters the population structure, and the age structure approaches spindle shape. The population structure is unstable and shows a declining trend. Disturbance affects the population structure and stability of *A. faxoniana*. Under light disturbance, the population structure is stable with high growth potential; the population grows steadily after passing through the bottleneck period. Under moderate disturbance, the population's growth potential declines. Under severe disturbance, there is a severe lack of young individuals, resulting in insufficient growth potential. Therefore, populations experiencing moderate to severe disturbance require enhanced protection to promote population recovery and stability. Our findings reveal the effect of different levels of disturbance on the population structure and regeneration of *A. faxoniana*. This provides a basis for restoring and regulating forest ecosystems in high‐altitude areas.

## Author Contributions

**Yang Zhao:** conceptualization (equal), data curation (equal), formal analysis (equal), investigation (equal), methodology (equal), visualization (equal), writing – original draft (lead). **Bo Li:** conceptualization (equal), data curation (equal), project administration (equal). **Wen Yu:** data curation (equal), software (equal). **Rui Qi:** investigation (equal), methodology (equal), resources (equal). **Benqiang Gao:** investigation (equal). **Xuemei Ha:** investigation (equal). **Ting Liu:** software (equal), visualization (equal). **Yi Li:** funding acquisition (supporting), resources (supporting).

## Funding

This study was supported by the Gansu Province Science and Technology Program (Grant No. 23YFFA0023), the National Natural Science Foundation of China (Grant No. 32260281), and the Gansu Province Forestry and Grassland Science and Technology Innovation Program (Grant No. KJCX202609).

## Conflicts of Interest

The authors declare no conflicts of interest.

## Supporting information


**Data S1:** ece373942‐sup‐0001‐DataS1.xlsx.

## Data Availability

All data supporting the findings of this study have been uploaded as Data S1.
